# TCM-Based Therapy as a Rescue Therapy for Re-Eradication of *Helicobacter pylori* Infection: A Systematic Review and Meta-Analysis

**DOI:** 10.1155/2022/5626235

**Published:** 2022-02-24

**Authors:** Mao-Feng Zhong, Jun Li, Xiao-Lin Liu, Peng Gong, Xiao-Tian Zhang

**Affiliations:** ^1^Department of Preventive Medicine, Shuguang Hospital, Shanghai University of Traditional Chinese Medicine, Shanghai 201203, China; ^2^Characteristic Diagnosis and Treatment Technology Research Institution Affiliated to Shanghai Institute of Traditional Chinese Medicine, Shanghai 201203, China; ^3^Department of Traditional Chinese Medicine, The 940th Hospital of Chinese People's Liberation Army, Gansu Province, Lanzhou 730050, China; ^4^Department of Gastroenterology, Shanghai Municipal Hospital of Traditional Chinese Medicine, Shanghai University of Traditional Chinese Medicine, Shanghai 201900, China

## Abstract

The increase in drug-resistant strains poses a severe challenge for *Helicobacter pylori* (Hp) treatment, and the failure of traditional triple or bismuth quadruple therapy makes it difficult to eradicate Hp. Tailored therapies should be expanded, and traditional Chinese medicine (TCM) may provide the potential regimen. The aim of the present study is to systematically compare TCM-based therapy (TCM combined with Western medicine) and Western medicine as a rescue therapy for Hp re-eradication. Studies through June 12, 2021, with keywords “*Helicobacter pylori*,” “medicine, Chinese traditional,” or “rescue treatment” and their related expressions were retrieved from PubMed, SinoMed, China National Knowledge Infrastructure, and Wanfang databases. Randomized clinical trials based on PICOS (population, intervention, comparators, outcomes, and study design) eligibility criteria that evaluated the efficacy and safety of integrated therapy on Hp re-eradication were included. The extracted contents included the demographic data of the participants, specific treatment measures, and the results of outcome indicators and safety indicators. Review Manager 5.3 software was used to perform this meta-analysis. Outcome measures including the HP re-eradication rate, symptom remission rate, and adverse effects were seriously analyzed. Under the guide of PRISMA, 18 studies were finally included. Pooled results showed significant differences in eradication rate between integrated and Western medicine therapy in intention-to-treat (ITT) analysis (OR = 2.21, 95% CI: (1.74, 2.81), *P* < 0.01). Symptom remission is higher in the administration of integrated therapy than in the administration of Western medicine therapy (OR = 2.45, 95% CI: (1.78, 3.37), *P* < 0.01). It is also indicated that integrated therapy showed significantly less adverse effects (OR = 0.60, 95% CI: (0.42, 0.84), *P* < 0.01. In conclusion, compared with Western medicine therapy, integrated therapy yields a higher eradication rate and acceptable safety profiles.

## 1. Introduction

*Helicobacter pylori* (Hp) is a flagellated Gram-negative and spiral-shaped bacterium that is colonized in the stomach, which is one of the most common chronic bacterial infections in humans, affecting approximately 4.4 billion individuals worldwide [1, 2]. In China, approximately 50% to 80% of the population is infected with Hp, and the rate is still rising [3]. Although most patients infected with Hp are clinically asymptomatic, it is responsible for gastrointestinal diseases such as chronic gastritis and peptic ulcers since it penetrates the mucosal layer of the upper gastrointestinal tract and causes chronic gastric inflammation [4]. On the contrary, classified as a Group 1 carcinogen by the International Agency for Research on Cancer, it is indicated that 1%–3% of the Hp-infected patients may develop gastric cancer [5]. Therefore, eradicating Hp may relieve Hp-related gastrointestinal diseases and reduce the risk of gastric cancer.

It is rarely hard for the spontaneous clearance of Hp once it colonizes in the stomach, so antibiotic therapy is recommended for the treatment of Hp. Triple therapy including clarithromycin, amoxicillin, and a proton pump inhibitor (PPI) is the standard worldwide [6]. However, the eradication rate decreased year by year due to bacterial factors, patient compliance, and reinfection; among which, antibiotic resistance is the most important factor [7] and resistance to clarithromycin is particularly serious. For patients who were treated with triple therapy yet failed, a bismuth quadruple therapy (such as metronidazole, tetracycline, bismuth, and a PPI) is recommended [8]. Nevertheless, the resistance rate of metronidazole and tetracycline has also exceeded 15% globally [9]. Besides, other regimens such as adding probiotic supplementation as an emerging therapy may also improve the eradication rate and alleviate side effects [10], but there is not enough evidence and consensus.

Traditional Chinese medicine (TCM) has attracted more and more attention because of its definite curative effect. Although there is no record of Hp in TCM, TCM has a long history in the treatment of various gastric diseases which are related to Hp. In ancient Chinese medicine, there were many monographs, such as Li Gao's Treatise on the Spleen and Stomach, focusing on the treatment of spleen and stomach diseases. Therefore, TCM has accumulated rich experience and achieved satisfactory curative effects in the treatment of Hp-related diseases, which indicates that many Chinese herbs have the potential effect of re-eradicating Hp. Holism and syndrome differentiation and treatment are the root concepts of TCM. Recipe composed of various herbs and individual mode of herbs use makes it difficult to cause drug resistance in Hp and it may be an excellent candidate rescue therapy for re-eradication of Hp. More and more studies in recent years also showed that many TCM decoctions, such as Banxia Xiexin Decoction, combined with Western medicine have significant effects on the treatment of Hp. Chinese herbs such as Huangqin (*Radix Scutellariae*) and Huanglian (*Rhizoma Coptidis*) have a certain inhibitory effect on Hp [11, 12]. However, there is no solid evidence to support a consensus on TCM as a rescue therapy for re-eradication of Hp at present, due to the differences among clinical trials in terms of research program and efficacy. Therefore, we perform a meta-analysis to evaluate Hp re-eradication rates of triple/quadruple therapy combined with TCM and hope to promote the development and utilization of TCM to a greater extent.

## 2. Methods

### 2.1. Search Strategy

The following databases were searched from their inception through June 12, 2021, for studies matched our search strategy: PubMed, SinoMed, China National Knowledge Infrastructure, and Wanfang databases. The following keywords were searched: “*Helicobacter pylori*” and “medicine, Chinese traditional” or “integrative medicine” or “plants, medicinal” or “drugs, Chinese herbal” and “remedial treatment” or “rescue treatment” or “salvage treatment”. The search languages were limited to English and Chinese. In addition, the references of eligible studies, related books, conference articles, and protocols were also manually searched to provide a comprehensive review.

### 2.2. Study Selection

We followed the Preferred Reporting Items for Systematic Reviews and Meta-analyses (PRISMA) guidelines [13] for this systematic review and meta-analysis. The included studies were selected based on PICOS (population, intervention, comparators, outcomes, and study design) eligibility criteria. The inclusion standards were as follows: (a) subjects with Hp-positive and without region, sex, and age restrictions; (b) both control group (defined as patients treated with only Western medicine) and experimental group (defined as patients treated with TCM combined with Western medicine) have received the first-line therapy regimens [8] recommended in the current Consensus Report and failed to eradicate Hp; (c) TCM treatments in the experimental group include single herb, formulas, or herbal products in any formulations such as oral decoction, pill, powder, or granules; (d) randomized controlled and parallel trials were performed, regardless of whether blinding was adopted, and had a duration of at least one week; and (e) the trial included outcome indicators with Hp eradication rates at least. The definition of the Hp eradication rates must conform to the requirements of the current Consensus Report.

Exclusion criteria were as follows: (a) subjects with bleeding ulcers or gastric cancer; (b) other TCM treatments such as acupuncture and diet therapy; and (c) data details such as conference abstracts that could not be obtained.

Two researchers determined whether the articles met the inclusion standards independently. All publications were managed by EndNote X7 software (Thomson Scientific Ltd., Canada). Discrepancies between researchers were resolved by a third researcher.

### 2.3. Data Collection and Assessment

We performed the specialized spreadsheets to collect the extracted data. The extracted contents included the demographic data of the participants, specific treatment measures, and the results of outcome indicators and safety indicators. The primary outcome was the HP re-eradication rate, while the secondary outcome was the symptom remission rate. The outcome indicators are listed in the summary of findings table and reasons for downgrading were provided by adhering to the GRADE guidelines. If there were disagreements between researchers, a third researcher would try to achieve a consensus.

### 2.4. Quality Appraisal

Cochrane Collaboration's risk of bias assessment tool was applied to appraise the risk of bias of each included study. The tool focuses on the study randomization method, allocation concealment, blinding, outcome measures including data completeness and follow-up, and potential sources of bias.

### 2.5. Statistical Analysis

All statistical analyses were conducted with RevMan 5.3 software. Continuous data were performed as mean values and standard deviations, while odds ratios and 95% confidence intervals (CI) were reported for the dichotomous outcomes. Sensitivity analysis was performed by deleting one study at a time to reflect the influence of the individual outcomes on the overall outcome. Heterogeneity was tested using a standard chi-square test and *I*^2^ statistic, and the random-effects model was applied when *I*^2^ > 50% without clinical heterogeneity; otherwise, the fixed-effects model or subgroup analysis based on treatment type, clinical heterogeneity, etc., was used. Two-tailed *P* < 0.05 was considered statistically significant. Forest plots demonstrated effect estimates of the included trials visually. The publication bias risks were assessed by funnel plots.

## 3. Results

### 3.1. Search Results

**Figure 1:**
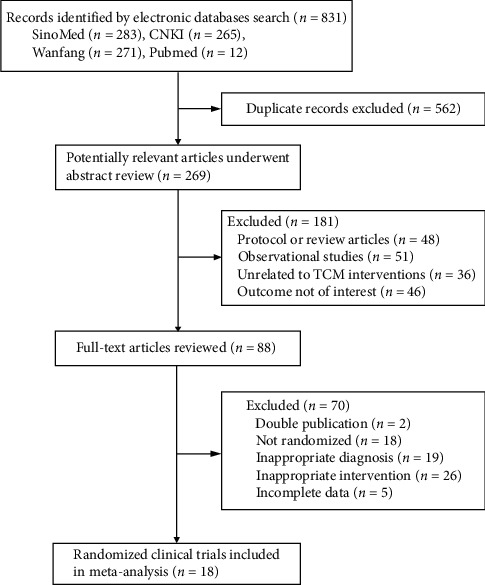
PRISMA flow diagram of articles included in the meta-analysis.

Under the guidance of PRISMA, 831 articles were achieved from all the databases mentioned above. However, there were 562 duplicate records, and after reviewing the abstract, further 181 studies were excluded since they were protocol, review, or observational studies that did not meet our enrolled criteria. By reviewing the full text of the remaining studies, 70 studies were excluded due to methodological weaknesses such as not randomized or incomplete data. Finally, eighteen studies were included for further analysis in the present study (Figure 1).

### 3.2. Characteristics of the Included Studies

Characteristics of the included studies are shown in Table 1. Overall, these 18 trials were reported between 2005 and 2020 that included 1990 participants whose mean age was 43.62 years, with 1001 female subjects, and the range of size of trials was 56 to 187 participants. Only one trial [30] was a multicenter RCT and the others were single-center trials. All the enrolled subjects performed urea breath test or rapid urease test initially and after 4 weeks to confirm the eradication of Hp. Chronic gastritis and peptic ulcer were the most common accompanying diseases. Most of the interventions in the experimental group were treatment of the control group combined with TCM such as Jinghua Weikang capsule and herb mixture. However, several studies compared the TCM with specific drug in the control group. For example, Zhang et al. [30] replaced bismuth potassium citrate (BPC) in the control group with Jianwei Qingyou decoction in the experimental group. Intention-to-treat (ITT, 14 trials) analysis, per-protocol (PP, 14 trials) analysis, or both analyses (10 trials) were applied to assess the eradication rates. The eradication rates range from 74.40% to 94.00% in the experimental group while from 66.67% to 85.00% in the control group with ITT analysis, and PP analysis showed the same trends.Age was expressed as mean ± standard deviation. E/C: experimental group/control group; NA: not available; CLA: clarithromycin; BPC: bismuth potassium citrate; CBD: colloidal bismuth subcitrate; PPI: proton pump inhibitor; FZD: furazolidone; LVFX: levofloxacin; ITT: intention-to-treat; PP: per protocol; ①: Hp eradication rate; ②: percentage of adverse effects; ③: liver and kidney function tests; ④: clinical symptoms improved conditions.

### 3.3. Quality Assessment and Risk of Bias

**Figure 2:**
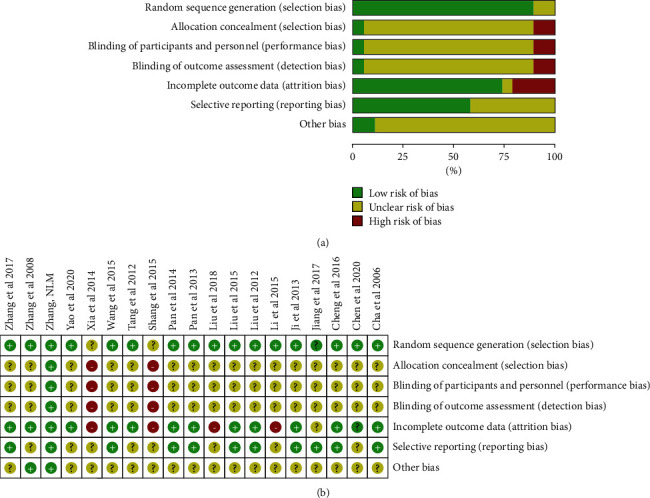
Quality assessment of the enrolled studies in this analysis. (a) Risk of bias for all the included studies. (b) Risk of bias for each included study.

Using Cochrane's Risk of Bias assessment tool, the risk of bias for all studies is shown in Figure 2. Random sequence generation was assessed as an unclear risk of bias in two studies [18, 24]. None of the trials mentioned a placebo for Chinese herbal medicines since blinding of researchers and participants during the distribution of herbal formulas was not possible. Allocation concealment and detection bias (blinding of outcome assessment) displayed an unclear risk of bias in all studies. None of these studies clearly reported allocation concealment or methods for blinding outcome assessments, which was the main reason for the judgement of an unclear risk of bias. Seven studies did not report adverse effects after medication [15, 16, 20–22, 30, 31], which would increase the risk of attrition bias (incomplete outcome data). Because the Hp clearance rate is the key outcome indicator, which had been statistically detailed in all studies, reporting bias (selective reporting) and other sources of bias were considered to be low.

### 3.4. Eradication Rate of Integrated Therapy versus Western Medicine Therapy

**Figure 3:**
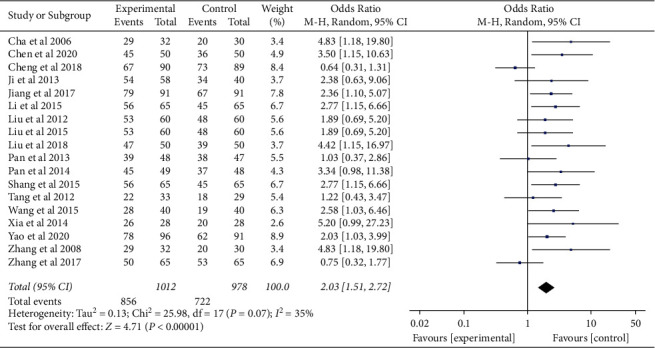
Comparison of all the enrolled studies for Hp re-eradication rate.

With ITT analysis for the enrolled studies, significant heterogeneity in the consistency of the trial results was shown (*P* = 0.07; *I*^2^ = 35%). Therefore, a fixed-effects model was used for statistical analysis. The odds ratios of integrated therapy versus Western medicine therapy in improving the Hp clearance rate was 2.03 (95% CI (1.51, 2.72)) (Figure 3). In order to increase the credibility of the result, the trial [30] with distinct grouping method was excluded and the remaining 17 trials showed no heterogeneity in the consistency of the trial results (*P* = 0.46).

**Figure 4:**
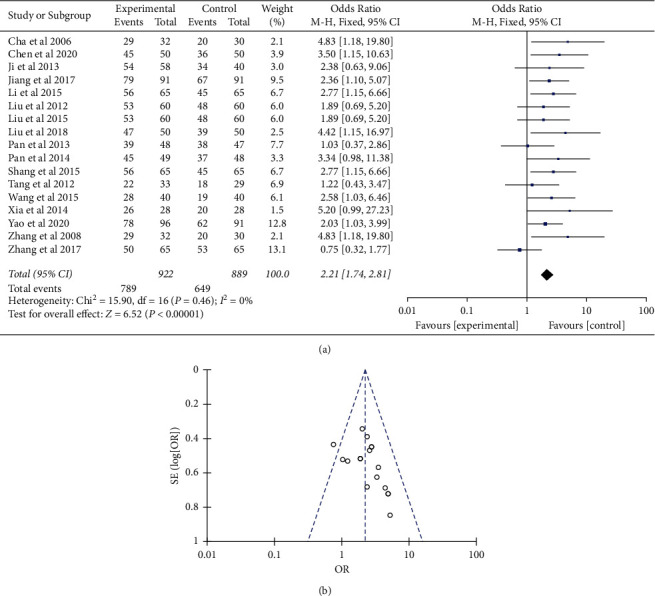
Comparison of the integrated therapy *vs.* Western therapy for Hp re-eradication rate after excluding one study. (a) Forest plot of comparison of the included trials. (b) Funnel plot of comparison of the included trials.

Compared with Western medicine therapy, integrated therapy showed favored eradication rate with odds ratios in improving the Hp clearance rate of 2.21 (95% CI (1.74, 2.81); *P* < 0.01) (Figure 4(a)). Sensitivity analyses suggested that the pooled effect of integrated therapy versus Western medicine therapy was not affected by changing effect model, and the funnel plot was symmetric (Figure 4(b)).

### 3.5. Symptom Remission of Integrated Therapy versus Western Medicine Therapy

**Figure 5:**
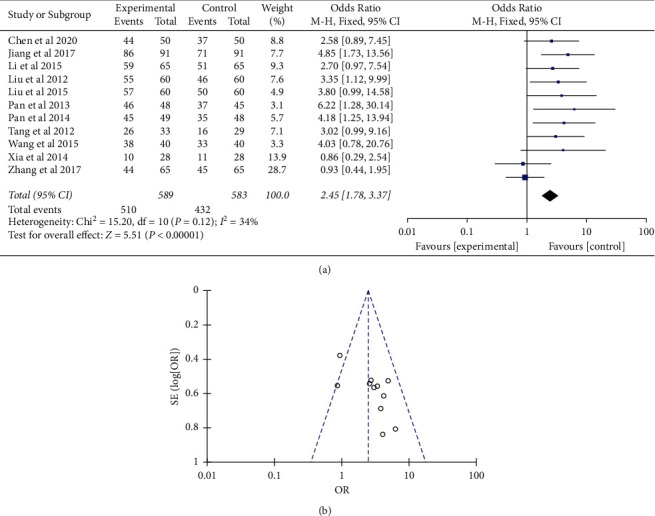
Comparison of the percentage of symptom remission between integrated therapy and Western therapy. (a) Forest plot of comparison of the included trials. (b) Funnel plot of comparison of the included trials.

Among the enrolled trials, 11 trials with 1172 patients had reported curative effect evaluation that were included in the statistical analysis [14, 15, 17, 18, 22–26, 28, 29]. There was mild heterogeneity in the consistency of the trial results (*P* = 0.12; *I*^2^ = 34%). Therefore, a fixed-effects model was applied for statistical analysis. The result showed that, compared with the Western medicine therapy, the odds ratio of the combination of integrated therapy for the curative effect was 2.45 (95% CI (1.78, 3.37)) (*P* < 0.01) (Figure 5(a)). Sensitivity analyses suggested that the pooled effect of integrated versus Western medicine therapy was not affected by changing effect model. The funnel plot was symmetric (Figure 5(b)).

### 3.6. Safety Outcomes of Integrated Therapy versus Western Medicine Therapy

**Figure 6:**
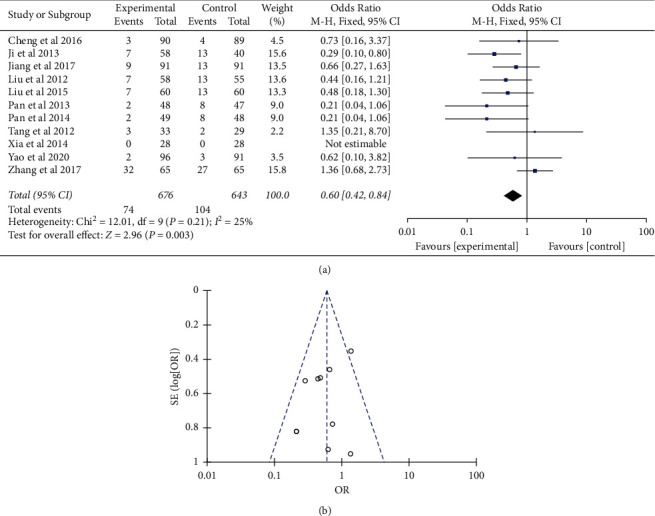
Comparison of the percentage of adverse effects between integrated therapy and Western therapy. (a) Forest plot of comparison of the included trials. (b) Funnel plot of comparison of the included trials.

The safety assessment of drugs is another important aspect for treatment regimen formation. In this study, 11 trials with 1263 patients had reported the adverse reactions that were included in the statistical analysis [14, 17, 18, 23–30]. There was mild heterogeneity in the consistency of the trial results (*P* = 0.21; *I*^2^ = 25%). Therefore, a fixed-effects model was used for statistical analysis. Results indicated that the odds ratio of the integrated therapy for the percentage of adverse effects was 0.60 when compared to Western medicine therapy (95% CI (0.42, 0.84)) (*P* < 0.01) (Figure 6(a)). Sensitivity analyses suggested that the pooled effect of integrated versus Western medicine therapy was not affected by changing effect model. The funnel plot was symmetric (Figure 6(b)). Dizziness, nausea, and diarrhea were the most common adverse effects which were reported in 9 trials, followed by rashes that were reported in 6 trials and fatigue, constipation, and abdominal pain in 3 trials. Besides, one trial observed no side effects in either group [24]. No serious adverse events were reported.

## 4. Discussion

Hp treatment is an important global issue since it may cause several gastrointestinal diseases. Although about 85% of Hp-infected patients are asymptomatic, the remaining 15% may have a chance to develop peptic ulcer disease and about 1% may finally develop gastric cancers [32]. The increase in drug-resistant strains poses a severe challenge for Hp treatment, and the failure of traditional triple therapy makes it difficult to eradicate Hp. Rescue therapy such as bismuth quadruple therapy may be effective at first, but the rate of antibiotic resistance of Hp is increasing over time. Therefore, tailored therapies should be expanded and TCM may provide the potential regimen.

In recent years, with the modernization of TCM research progress, parts of the Chinese herbs compound, single drug, or their extracts with a clear effect on the treatment of Hp infection have been gradually excavated. However, TCM is still not a mainstream treatment so that the effect of its application alone cannot be evaluated. However, there have been a lot of clinical studies which set TCM as a supplement to the existing Western medicine treatment and showed perfect effect, although no consensus has been reached. Therefore, the present study tries to compare the effects between integrated and Western medicine alone and tries to clarify whether TCM has the advantage of strengthening the existing Western medicine therapy.

In this meta-analysis, the pooled data exhibited a better significant therapeutic value in TCM-based therapy than Western drugs alone. According to the eradication grade of the Hp eradication efficacy (eradication rate of >95% is excellent, 90–95% is good, 85–89% is fair, 81%–84% is bad, and <80% is unacceptable), in our ITT analysis, 6 reports showed that TCM-based therapy yielded a good eradication rate, 5 reports showed TCM-based therapy yielded a fair eradication rate, 1 and 2 reports showed that TCM-based therapy yielded a bad and unacceptable eradication rate, respectively. On the other hand, no study showed that patients in the control group treated with Western drugs alone yielded a higher eradication rate than those in the experimental group treated with TCM combined with Western drugs, and 10 studies showed that Western drugs alone yielded an unacceptable eradication rate.

The identification of optimal regimens in the treatment of Hp is challenging due to increasing resistance to antibiotics. The Consensus Report recommends “PPI + bismuth +2 antibiotics” as the preferred choice for the first Hp eradication treatment. For those who failed to eradicate for the first time, it is recommended to take the susceptibility testing as a guide to re-treatment and comprehensively analyze the history of individual antibiotics, weigh the cost-effectiveness and the incidence of adverse reactions, and adopt sequential therapy or triple/quadruple therapy or combined probiotics and other new drugs. Given this clinical reality, TCM-based therapy may be an appropriate alternative treatment option for Hp infection.

Experimental and clinical studies have confirmed that the kind of clearing heat and detoxifying, resolving dampness to move qi, tonifying and replenishing the middle qi, activating blood and resolving stasis Chinese herbs can inhibit and kill Hp. Huanglian Jie Du decoction, Banxia Xiexin decoction, Xiang Sha Liu Jun Zi decoction, Zuojin Wan, Jinghua Weikang capsule, Jianwei granule, Binlang Sixiao tablet, etc., all have varying degrees of anti-Hp.

There are several limitations in this study. The number of included trials was still small, and the sample size was not large enough for the majority of trials. The available studies were of low quality; therefore, more well-designed studies are required to further confirm the findings. It is also hard to compare the effects of different TCM regimens to provide the best treatment for Hp re-eradication. Despite these limitations, our meta-analysis provides a better understanding of the benefit of TCM-combined therapy. The TCM-combined therapy showed superior efficacy to the empirical treatment in terms of Hp re-eradication. In addition, the TCM-combined therapy showed comparable tolerability and incidence of adverse events to those of the empirical treatment.

## 5. Conclusion

We highlight the advantages of using TCM in combination with Western medicine therapy as a rescue therapy for re-eradication of Hp, which yields a higher eradication rate and acceptable safety profiles when compared with Western medicine therapy alone. Therefore, this review is instructive for the treatment of drug resistance in Hp with integrated therapies and can be applied in practice.

## Figures and Tables

**Table 1:** Baseline characteristics.

Study	Study period	Total cases (E/C)	Age (E/C)	Male-female (E/C)	Accompanying diseases	Regimen		Duration (d) (E/C)	Previous treatment regimens	Adverse effects (E/C)	Outcomes	ITT eradication rates (%) (E/C)	PP eradication rates (%) (E/C)
						Experimental group	Control group						
Yao et al. [14]	2017.11–2019.01	96/91	51.3 ± 9.7/50.5 ± 10.9	36-60/37-54	Chronic gastritis; peptic ulcer	Control + Jinghua Weikang capsule 160 mg, tid	Esomeprazole 20 mg + amoxicillin 1.0 g/furazolidone 0.1 g + CLA 0.5 g + BPC 220 mg, bid	14/14	CLA triple therapy	2/3	①②④	NA	83.90/71.30
Chen et al. [15]	2013.01–2017.12	50/50	36.8 ± 8.2/37.2 ± 7.8	23-27/20-30	Chronic gastritis	Control + Jianpi Qingyou decoction 200 mL, bid	CBD 0.2 g + rabeprazole 10 mg + CLA 0.5 g + amoxicillin 1.0 g, bid	14/14	PPI triple therapy	NA	①④	NA	93.80/73.50
Liu [16]	2015.03–2016.03	50/50	32.1 ± 16.5/35.6 ± 15.4	32-18/35-15	Chronic gastritis; peptic ulcer	Control + Sijunzi formula 100 mL, bid+	Bismuth quadruple therapy	7/14	Bismuth quadruple therapy, 14 d	NA	①	94.00/78.00	NA
Zhang et al. [17]	2015.01–2016.04	65/65	41.9 ± 11.0/44.9 ± 10.8	35-30/31-34	NA	Berberine 0.3 g, tid + esomeprazole 20 mg + CBD 220 mg, bid;	Esomeprazole 20 mg + CBD 220 mg + tetracycline 750 mg + FZD 0.1 g bid	14/14	CLA + amoxicillin + PPI + bismuth, 14 d	6/4	①②④	76.90/81.50	84.70/86.90
Jiang et al. [18]	2014.01–2015.10	91/91	49.6 ± 13.2/48.5 ± 11.6	43-48/51-40	Peptic ulcer; chronic gastritis	Control + Qi'e decoction 1#, bid	Esomeprazole 20 mg + FZD 0.1 g, bid; LVFX 0.5 g, qd; *Bacillus subtilis* enterococcus capsule 0.5 g, tid	14/14	Standard triple therapy or bismuth quadruple therapy	9/13	①②④	86.80/73.60	89.80/78.80
Cheng et al. [19]	2013.01–2014.12	90/89	48.0 ± 13.0/49.0 ± 12.0	37-53/36-53	NA	Control + Jinghua Weikang capsules 240 mg, bid	BPC 220 mg + pantoprazole 40 mg + amoxicillin 1.0 g + FZD 0.1 g, bid	10/10	NA	3/4	①②	74.40/82.00	76.10/85.90
Shang et al. [20]	2011.01–2013.04	65/65	37.8 ± 3.3/37.3 ± 3.2	41–24/39–65	Peptic ulcer; duodenal ulcer; gastric ulcer	Control + Chinese herbs 200 mL, bid	CLA 0.5 g + rabeprazole 20 mg + metronidazole 0.4 g, bid	NA	CLA + rabeprazole + amoxicillin	NA	①	86.15/69.23	NA
Li [21]	2011.01–2012.04	65/65	37.8 ± 3.3/37.3 ± 3.2	41-24/39-26	Duodenal ulcer; peptic ulcer; gastric ulcer	Control + Chinese herbs 300 mL, bid	Amoxicillin 0.5 g + LVFX 0.2 g, bid; rabeprazole 20 mg, qd	NA	CLA + rabeprazole + metronidazole	NA	①②	86.15/69.23	NA
Wang et al. [22]	2014.01–2015.01	40/40	42.3 ± 12.2/44.8 ± 12.0	17-21/20-17	NA	Chinese herbs 150 mL, bid for 28 days + amoxicillin 1 g bid + LVFX 0.5 g qd + rabeprazole 20 mg bid for 10 days;	Amoxicillin 1 g bid + LVFX 0.5 g qd + CBD 150 mg tid for 10 days; rabeprazole 20 mg bid for 18 days	28/18	Standard triple therapy	NA	①④	NA	73.68/51.35
Liu and Liu [23]	NA	60/60	46.3 ± 11.4/46.8 ± 12.2	21-39/22-38	NA	Control + Binlang Sixiao tablet 2.4 g, bid	Rabeprazole 10 mg + CBD 240 mg + amoxicillin 1.0 g + FZD 0.1 g, bid	14/10	NA	7/13	①②③④	88.33/80.00	91.38/87.27
Xia [24]	2011.06–2013.06	28/28	39.6 ± 5.3/38.2 ± 4.7	20-8/19-9	NA	Jinghua Weikang capsule 160 mg, tid for 4 weeks + moxifloxacin 0.4 g qd + amoxicillin 0.5 g + rabeprazole 10 mg bid	CLA 0.5 g + amoxicillin 0.5 g + rabeprazole 10 mg, bid	14/14	NA	0/0	①②④	92.80/71.40	NA
Pan et al. [25]	2011.04–2013.04	49/48	43.1 ± 12.7/43.9 ± 14.4	28-21/25-23	Chronic gastritis; peptic ulcer	Jiawei Zuojin pill + sequential therapy of esomeprazole 20 mg + amoxicillin 1.0 g + FZD 0.1 g, bid	Esomeprazole 20 mg + CBD 0.3 g + amoxicillin 1.0 g + FZD 0.1 g, bid	10/10	NA	2/8	①②④	91.80/77.10	93.80/82.20
Pan et al. [26]	2011.01–2013.01	48/47	43.4 ± 12.1/44.3 ± 12.4	29-19/26-21	Chronic gastritis; peptic ulcer	Zuojin pill + esomeprazole 20 mg + amoxicillin 1.0 g + FZD 0.1 g, bid	Esomeprazole 20 mg + CBD 0.3 g + amoxicillin 1.0 g + FZD 0.1 g, bid	10/10	NA	2/8	①②④	81.30/80.90	84.80/86.40
Ji et al. [27]	2008.03–2010.11	58/40	42.3 ± 8.3/40.5 ± 10.1	37–21/26–14	Chronic gastritis; gastric ulcer; duodenal ulcer	Control + Changzhong capsule 5#, tid	Omeprazole 20 mg + amoxicillin 1.0 g, bid + LVFX 0.5 g, qd	20/7	Standard triple therapy	7/13	①②③	93.00/85.00	95.00/89.00
Liu [28]	NA	60/60	46.5 ± 11.7/46.3 ± 11.4	21-49/22-38	NA	Control + Chinese herbs 200 mL, bid	Esomeprazole 20 mg + BPC 0.6 g + amoxicillin 1.0 g + FZD 0.1 g, bid	14/7	NA	7/13	①②③④	88.33/80.00	91.38/87.27
Tan et al. [29]	2010.08–2012.04	33/29	NA	NA	Gastric ulcer; duodenal ulcer; chronic gastritis	Jinghua Weikang capsule 160 mg, tid + rabeprazole 10 mg + LVFX 0.2 g + FZD 0.1 g, bid	Rabeprazole 10 mg + amoxicillin 1.0 g + moxifloxacin 0.4 g, bid + BPC 1.3 g, tid	7/7	Standard triple therapy	3/2	①②④	NA	75.90/66.70
Zhang et al. [30]	2006.05–2007.11	32/30	42.5 ± 10.8/41.5 ± 11.3	17-15/14-16	Duodenal, gastric and peptic ulcer; chronic gastritis	Jianwei Qingyou decoction + omeprazole 20 mg + amoxicillin 1.0 g + CLA 0.5 g, bid	Omeprazole 20 mg + amoxicillin 1.0 g + CLA 0.5 g + BPC 220 mg, bid	7/7	Standard triple therapy	NA	①	90.60/66.67	93.50/71.40
Cha et al. [31]	2005.02–2005.12	32/30	42.5 ± 10.8/41.5 ± 11.3	17-15/14-16	Chronic gastritis; peptic ulcer	Jianwei granule 10 g, tid + omeprazole 20 mg + amoxicillin 1.0 g + CLA 0.5 g, bid	Omeprazole 20 mg + amoxicillin 1.0 g + CLA 0.5 g + BPC 220 mg, bid	7/7	Standard triple therapy	NA	①	90.60/66.67	93.50/71.40

## Data Availability

The data used to support the findings of this study are available from the corresponding author upon request.
